# Relationship Between Sedentary Lifestyle, Physical Activity and Stress in University Students and Their Life Habits: A Scoping Review with PRISMA Checklist (PRISMA-ScR)

**DOI:** 10.3390/brainsci15010078

**Published:** 2025-01-16

**Authors:** Mariasole Antonietta Guerriero, Anna Dipace, Antonietta Monda, Antonella De Maria, Rita Polito, Giovanni Messina, Marcellino Monda, Marilena di Padova, Angelo Basta, Maria Ruberto, Emanuele Capasso, Fiorenzo Moscatelli, Pierpaolo Limone

**Affiliations:** 1 Department of Humanistic Studies, University of Foggia, 71121 Foggia, Italy; mariasole.guerriero@unifg.it (M.A.G.); marilena.dipadova@unifg.it (M.d.P.); angelo.basta@unifg.it (A.B.); 2 Department of Psychology and Education, Pegaso Telematic University, 80143 Naples, Italy; anna.dipace@unipegaso.it (A.D.); pierpaolo.limone@unipegaso.it (P.L.); 3 Department of Human Science and Quality of Life Promotion, San Raffaele Telematic University, 00166 Rome, Italy; antonietta.monda@uniroma5.it; 4 Department of Education and Sport Sciences, Pegaso Telematic University, 80143 Naples, Italy; antonella.demaria@unipegaso.it (A.D.M.); maria.ruberto@unipegaso.it (M.R.); 5 Department of Psychology and Health Sciences, Pegaso Telematic University, 80143 Naples, Italy; rita.polito@unipegaso.it; 6 Department of Experimental Medicine, Section of Human Physiology and Unit of Dietetics and Sports Medicine, University of Campania “Luigi Vanvitelli”, 80138 Naples, Italy; giovanni.messina@unicampania.it (G.M.); marcellino.monda@unicampania.it (M.M.); 7 Department of Advanced Biomedical Sciences, University of Napoli “Federico II”, 80138 Naples, Italy; emanuele.capasso@unina.it

**Keywords:** physical activity, physical exercise, stress, mental well-being, perceived stress, university students, college students

## Abstract

The global prevalence of sedentary lifestyles and the associated health risks, such as cardiovascular, metabolic and mental issues, is an epidemic worldwide problem, particularly in the university population. Background/Objectives: University students are particularly vulnerable because of academic pressure and lifestyle changes. Despite the well-known benefits of physical activity in reducing mental stress and improving physical and mental well-being, the literature lacks effective interventions and standardized protocols for this population. This study aims to investigate the state of the art in literature regarding the correlation between the use of physical activity as a means of prevention and intervention and the effects on university students’ stress and mental well-being. Methods: A scoping review was conducted using the PRISMA protocol for scoping reviews, targeting university population with specific terms. Results: The review analyzed 61 articles and identified a consistent positive correlation between physical activity and stress reduction. Interventions included yoga, tai chi, aerobic exercise and moderate- or high-intensity exercise. A lack of standardized protocols were also evident. Conclusions: Physical activity is an effective means for managing stress and improving mental well-being among university students. An integrated approach combining different forms of exercise and strategies to regulate emotions could provide very effective effects on the mental well-being of students. Universities should propose physical activity programs in several forms to enable students to choose the most appropriate one and keep them active.

## 1. Introduction

The increasingly significant and constant spread of sedentary lifestyles and the sedimentation of such lifestyles are reaching alarming figures. Sedentary living is a child of the modern world which brings high risks. for the cardiovascular, organic and mental health of the population, exposing them to the risk of non-communicable diseases such as cancer, type 2 diabetes, heart and metabolic diseases [1,2]. The global prevalence of sedentary lifestyles, which involves between 60 and 85 percent of the population, carries a 20 percent higher risk of mortality than those who regularly engage in physical activity, and this is worsened by work and academic life that requires many hours of sitting between study and office work [3].

For this reason, even those who comply with the WHO guidelines (150–300 min of moderate-intensity aerobic physical activity; at least 75–150 min of vigorous-intensity aerobic physical activity) and spend the rest of the time inactive and not engaged in energy-consuming activities are still sedentary subjects overall [3]. Physical inactivity is understood as lack of physical activity according to the WHO recommendations [2]. Sedentary behavior and habits, stress and overweight resulting in obesity are among the main reasons for deaths and premature diseases in all age groups, including the youngest [4].

### 1.1. Sedentariness, Physical Activity and Mental Health

In addition to the well-known and dangerous risks on a metabolic and organic as well as structural level, sedentariness and prolonged physical inactivity are also a risk for the development or worsening of states of mental malaise and detriment to well-being on a psycho-physical level, leading to high rates of stress and symptoms of anxiety and depression [5]. Many studies in literature report how incorrect lifestyle attitudes often occur in coexistence [2], and this leads to an overlapping of risk factors and a more pronounced exposure to harmful consequences for mental and physical health.

### 1.2. University Students and Lifestyle Habits

Within this framework, there is a population at high risk, which is that of university students. University students, future adults, are a particularly fragile segment of the population and exposed to high health risks. In fact, their lives in the transition from adolescence to adulthood undergo a transitional phase that goes hand in hand with the transition from school to university [1]. Among the difficulties faced, especially for students living far from home, is a new management of life, autonomy, finances and one’s relationships to which academic stress and pressure is added [1,3,6]. As a result, university students are prone to psychological problems such as depression, anxiety and stress and adopt habits that are harmful to their health such as alcohol abuse and smoking.

A global estimate indicates a prevalence of depression and anxiety symptoms of 33.6% and 39.0%, respectively, among university students in 64 studies [7]. Approximately one third of university students suffer from at least one chronic illness, while recent studies indicate that a similar proportion struggle with psychological difficulties, suggesting that the university experience can be a significant source of stress, with students experiencing higher levels of anxiety and tension than their non-college peers, with negative effects on overall well-being [8].

The lifestyle habits of university students are sedimented during the university period and create what will be future lifestyle habits as well as attitudes toward physical activity and lifestyle [2,3,6].

Within this framework, between sedentariness and its risks and the lifestyle habits of university students, there is a strong means of prevention and intervention represented by physical activity [9].

### 1.3. The Role of Physical Activity

Physical activity, in a diametrically opposed way to sedentary activity, has several benefits that are well known in the literature. These include reduced risk of cardiovascular disease, improved cognitive and sleep function and reduced stress [10]: low levels of stress and anxiety are recorded particularly in students who achieve the WHO recommendations in terms of moderate- to vigorous-intensity physical activity [1]. It has been seen in the literature that strength activity, and in particular vigorous-intensity activity, has particularly high benefits in terms of physical, organic and mental well-being in university students. In university students, many health protection factors are in decline, such as muscle strength and cardiorespiratory capacity, which are trained with regular physical activity [4,6]. Choices in terms of exercise and physical activity in college may strongly impact future approaches to physical activity and may provide not only benefits but the structuring of long-term healthy and active lifestyle habits [5]. In addition, physical activity not only drastically reduces health risks but also has the effect of improving and enhancing academic success and university learning outcomes [8,10]. What is not clear from the literature, however, is which type of exercise or physical activities are most suitable to ensure the best possible long-term health benefit for students. Considering this alarming picture and considering that the university population is a vulnerable and fragile population due to the pressure they are under, making them more likely to adopt unhealthy lifestyles in managing themselves, and considering the stress and stress management burden students face, with the risk of incurring mental health problems, prompted this contribution.

### 1.4. Objective of the Study

The aim of this study is to investigate the state of the literature regarding the correlation between the use of physical activity as a means of prevention and intervention and the effects on university students’ stress and mental well-being by analyzing their lifestyles and sedentary levels, identifying the benefits, and assessing the presence of standardized intervention protocols in the literature.

### 1.5. Research Questions

With reference to the work carried out in this study, the following research questions were asked:

- RQ1: Does the literature present studies directly correlating physical activity, exercise and stress in the university population?
- RQ2: Can physical activity be considered as a means of promoting health and improving the state of stress and mental ill health in the university population?
- RQ3: Are there standardized intervention models or protocols in the literature for the university population whose results are generalizable?

## 2. Materials and Methods

**Figure 1:**
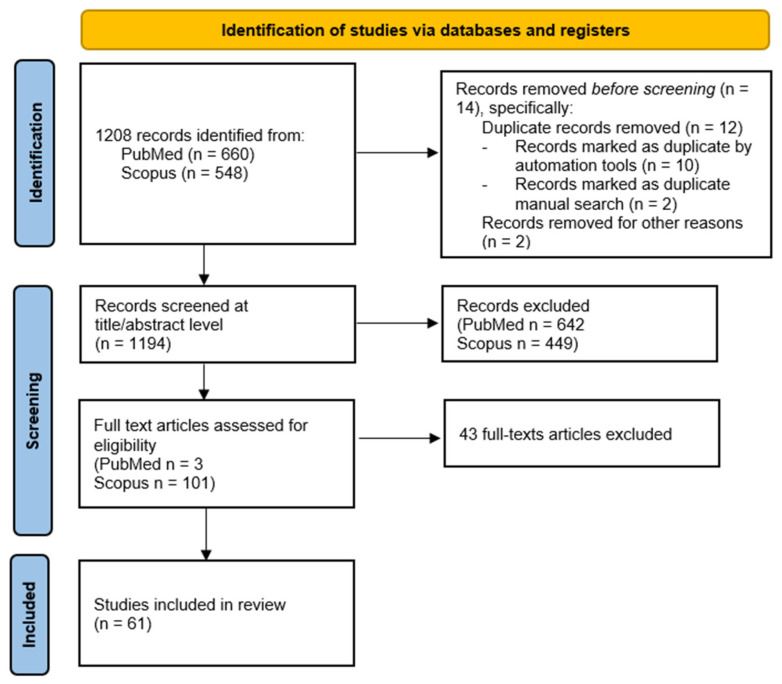
PRISMA flow chart of the conducted search. The figure illustrates a PRISMA flow diagram used to document the systematic review process. It shows the identification, screening, and inclusion stages of studies. Initially, 1208 records were identified from PubMed (660) and Scopus (548), with 14 duplicates and irrelevant records removed. After screening the titles and abstracts of 1194 records, 1091 were excluded. Of the 104 full-text articles assessed for eligibility, 43 were excluded, leaving 61 studies included in the final review.

This scoping review adheres to the Protocol for Scoping Review [11] using the Prisma Extension for Scoping Reviews Checklist (Figure 1), and a selection of articles in the literature was made using these guidelines. The checklist is available in Appendix B attached to this contribution. The research was conducted on two databases, Scopus and PubMed, between September and October 2024. The following search string and search terms were used for the research: physical activity OR physical exercise OR exercise AND university student OR college student OR university population OR undergraduate student AND stress OR stress level OR cortisol. The string has been limited to the presence of search terms in the abstract, title and keywords. The selected date range for the inclusion of articles was 2022–2024. Only open-access articles in English were considered. The focus was on magazine articles and a target population aged 19–44. All the papers resulting from the search have been included and exported to Zotero. The search produced 1208 results, which after removing duplicates and withdrawn publications have been reduced to 1194.

### 2.1. Identification Phase

In this phase, a search was carried out according to the above-mentioned methods, and 1208 results were obtained. The results were independently searched and analyzed but not blindly by two reviewers who then discussed the reasons for including or excluding the results. Only results deemed eligible by both parties were included. A total of 1194 results were included.

### 2.2. Screening Phase

**Figure 2:**
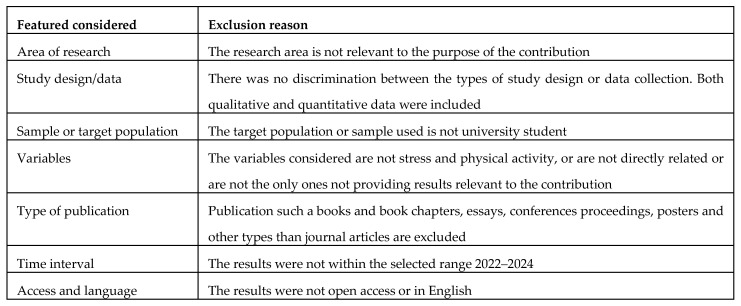
Exclusion criteria in the screening phase at the abstract and title level.

At this stage, articles have been included or excluded based on their titles or the content of their abstracts. The reasons for exclusion are shown in Figure 2. The criteria used for article selection were as follows: research area, study design or data collection, sample and target population, variables identified, type of publication, time interval of results found, access to papers, and language used. Regarding the research area, only papers relevant to the purpose of the contribution were considered; no distinction was made of the disciplinary field (health, medical, physiotherapy) as much as the relevance of the topic of the paper starting from the title and abstract. Regarding study design or data collection, all types of studies and all data collection methodologies were included if data collection was present. The sample or target population had to be represented only by the university population, so papers that contained references to university students and not to young adults in general were selected. Even in papers with the 19+ population, those that explicitly focused on the university population as the only target population were then selected and included in the next steps. The variables identified and whose possible relationship was seen in the literature were sedentariness in college students, levels and quality of mental health, and use of physical activity. Specific physical activity protocols were not selected as much as any regular and consistent motor, sport or daily activity with energy expenditure and according to WHO recommendations. The type of publications included choosing only scientific and journal articles, excluding essays, conference proceedings, abstracts and posters. The date range in which the results were considered and selected was in the range between 2022 and 2024, not taking into consideration papers prior to 2022. Finally, the articles included in the first selection phase were only in open access with full text available and in English, excluding papers that did not meet these criteria at the outset. The 105 results obtained were the result of an initial single selection based on the title and analysis of the abstracts with reference to the conditions described above. Results that were deemed correctly responsive to the first stage of selection by both reviewers were, therefore, retained. 

### 2.3. Inclusion Phase

Inclusion criteria, used for the final selection of suitable papers for the present study, were implemented during the phase following the analysis of abstracts with the analysis of the entire contribution. At this stage, the included studies had already been selected by the research area, target population, language, age group, and open access. We also excluded studies on COVID-19 as well as clinical or pathological populations or mental disorders such as anxiety, depression, post-traumatic stress disorder and the like. Thus, studies that dealt with the relationship between stress, physical activity, and the university population were included as well as studies that dealt with stress and mental well-being with physical activity understood as exercise; studies that dealt with mental well-being and/or stress and sports activity; and studies that dealt with mental well-being and/or stress in relation to increased physical activity understood as increased levels of daily movement. No distinction was made regarding physical activity, including all that had as its goal an effect on college students’ stress and well-being through movement practices and increased levels of physical activity or the reduction in sedentary levels. The inclusion phase produced a result of 61 articles.

## 3. Results

The search produced 61 results that met the reported inclusion criteria. The 61 were analyzed into the following elements to be able to classify them into macro-categories: scope of the research conducted, target population and reference institution, study design and thus type of analysis/research, type of physical activity.

At the thematic level, a single thread emerged in the theme of the use of physical activity, exercise and sport for stress management and the improvement of stress states and mental well-being. The two categories into which the results can be divided are literature studies (systematic and scoping reviews, literature or narrative reviews, etc.) and applied intervention protocols or questionnaire administration (Table 1).

Furthermore, studies that have included an intervention protocol can be distinguished according to the type of exercise administered. The most frequent physical activities that have emerged from studies are yoga, Taichi, aerobic activity, strength and resistance exercises at moderate to vigorous intensity. Moreover, studies included papers, and their objectives are resumed in the Appendix A. The results were analyzed in full by both reviewers. For experimental, quasi-experimental, and RCT studies, a qualitative analysis of internal study quality was performed by both reviewers using the PEDro scale. The score for each outcome analyzed is given in Appendix C.

## 4. Discussion

The university period is a transitional phase from adolescence to adulthood, moving from a simple lifestyle to one with many responsibilities and autonomous self-management. University students cope with distance from family and loved ones, managing their own home alone and their own finances, and academic pressure [46]. The mental and physical health of university students are showing increasingly low values [51] with an increasing manifestation of chronic mental problems and high levels of stress. To address the stress of academic challenges, increasing physical activity may be one of the most effective strategies for students, because an increased level of physical exercise positively affects the psychosomatic well-being by acting on the neuroendocrine system and mitigating the negative effects of stress on health [43]. Other sources of negative emotions in university students include competition, social stress, emotional problems and financial strain [26].

The purpose of this study was to investigate whether the literature contains studies analyzing the correlation of activity and exercise with stress and whether appropriate protocols had been found to improve the mental and physical well-being of university students. The literature showed that all studies found a positive correlation between stress, mental well-being and physical activity, as also reported by some meta-analyses and systematic reviews analyzed. Body function and physical quality are positively correlated with mental health and its dimensions, such as cognition, emotion, personality and adaptation [51], and the positive impact of exercise on stress and mental state is evident from the studies with the proposed practices remaining highly variable [51].

### 4.1. Theoretical Studies and Reviews: A General Overview

Theoretical research provides a solid basis for understanding how physical activity affects stress management. In the systematic review by Donnelly et al. [28], the physical activities used in stress management and improvement and their effectiveness were analyzed; the results of the systematic review report that among the physical activities proposed in the literature, dance and Pilates have been found to have a drastic impact on stress and quality of life for students. Among other physical activity interventions, moderate- to vigorous-intensity physical activity such as running, yoga, and strength training were also found to be beneficial means of improving the psychological state of stress. Woodall et al.’s review [47] of the literature conducted over a 20-year period of conceptually based fitness and wellness (CBFW) courses to analyze the effectiveness of these courses on cognition, mental and physical health revealed that there is a strong correlation between attending these courses and improved physical activity and mental health; in particular, Woodall et al. saw from the literature that the use of these courses on physical activity and physical activity itself can also stimulate lifestyle changes using Change Behavior Techniques (CBTs). Herbert [27] in his research on how to improve the lifestyle and well-being of university students stated that moderate- to vigorous-intensity exercises, including practices such as yoga, can have a positive influence in alleviating symptoms of perceived stress and contributing to well-being. A 2024 meta-analysis by Huang [46] that analyzes the effectiveness of physical activity interventions on university students confirms not only that physical activity is a valid intervention element for students’ mental well-being but also its positive impact on stress and mental health in university students, and a variability of different interventions used emerges. Thus, the literature shows not only the positivity of using exercise to improve stress but also a variability of proposals. For example, Martin’s [16] systematic review of the use of yoga as a means of well-being as well as academic improvement shows that the most successful strategies benefiting students’ well-being were a combination of yoga and mindfulness practices, which could be adapted especially for students facing the first impact of life transition. This element can be considered for the individualization and customization of interventions according to personal propensities and thus consider the variability of proposals as an opportunity.

### 4.2. Some Results from Experimental Studies

From the literature of the articles selected in this scoping, studies with direct evidence aimed at testing the effectiveness and benefit of physical activity proposals on the mental well-being and stress management of students were also analyzed. Hachenberger et al. [52] conducted a study on 90 students, some of whom wore an accelerometer, and reported positive impacts already with light physical activities measured during the specific examination period, including a significant reduction in their perceived stress, which was very high. In the study conducted by Flood et al. [13], an application was developed to stimulate and motivate students to adopt active lifestyles by providing guidance on physical activity, sleep and sedentariness. This intervention reported an improvement in mental well-being and, most importantly, that adherence to the recommendations correlated with a reduction in stress. In a study by Gubareva from 2024 [53] conducted on students in the exam period observing certain well-being parameters in student athletes and non-athletes, software results showed that cardiovascular and psychophysiological parameter values were better in student athletes than in non-athletes, indicating greater resistance to stress than in those who do not engage in any physical activity or sport.

Gao et al. [22] conducted a trial using yoga and aromatherapy and reported improved sleep in the female university students involved, but they did not observe a direct effect on stress, which they derived as a secondary benefit of improved sleep quality. Suwannakul et al. [19] conducted a study of 44 overweight female students with Surya Namaskar yoga interventions for 8 weeks. The group that practiced yoga showed a significant reduction in perceived stress levels and in physical fitness, while there was little or no change in the control group.

An interesting work conducted by Reschke in 2024 [44] analyzed physical activity as a recovery factor in exam preparation sessions. The results reported that remedial activities, in particular physical activity, are effective in reducing the stress that accompanies the long pre-exam period and are appreciated by students.

### 4.3. Absence of a Standard Protocol

The analysis of this scoping reveals an important element that also answers the second research question posed in this study. The varied literature analyzed reports many different proposals for physical activity, exercise and sporting activity as well as different ways of proposing the same activities. This absence of a protocol limits the possibility of having a single standard intervention adapted to several samples to generalize results. On the other hand, this demonstrates that there is a transversality in the use of physical activity in its various forms and thus flexibility and adaptability to the demands of the population on which it is intervening.

Several studies, including those by Suwannakul [19], Zhu et al. [38] and Szmodis et al. [43], have explored various physical activities such as yoga, aerobic exercise and team sports, finding positive effects but with results that vary depending on the intensity, frequency and duration of the exercise. All activities reported in the literature showed benefits on stress, but while high-intensity sports such as running or weight-bearing activities reported significant benefits, other activities such as yoga or lower-intensity activities showed less pronounced, albeit present, impacts.

Yue and Xiao conducted a 2023 [54] study involving 100 students in at least 30 min of moderate-intensity physical activity for three months, including badminton. The positive effects reported were emotional recovery and improved resistance to physical exhaustion. Other moderate-intensity exercises with a beneficial effect on symptoms of stress and anxiety were identified by Herbert [16], who points out that walking or swimming are beneficial for managing states of mental malaise.

Brown et al. conducted a study in 2024 [20] using the 12-week PEAK intervention protocol (a CBT-based intervention protocol to improve exercise approach and motivation) involving moderate- to vigorous-intensity exercise. The results reported that improved strength and endurance training improved cognitive performance and emotional regulation.

Barradas et al. [48] also propose a combination of activities included in academic programs including aerobic physical activities with coping strategies and mindfulness. A good perspective for the future may be a comprehensive physical activity regimen integrating aerobic exercises and mindfulness as an approach for university students, helping them to manage both immediate stress and long-term physical and mental challenges.

In conclusion, the results of this scoping review confirm the beneficial effect of exercise, in any form, to manage, prevent and reduce stress in university students.

#### Research Questions

Summarizing the above findings of this paper, we report the answers to the three research questions posed at the beginning of the paper.

- RQ1: The literature showed that all studies found a positive correlation between stress, mental well-being and physical activity, as also reported by some meta-analyses and systematic reviews analyzed.
- RQ2: The results of this scoping review confirm and highlight the beneficial effects of any form of physical activity and movement to manage, prevent and reduce the effects of stress.
- RQ3: The results of this scoping review emphasize the lack of a specified and standardized protocol

### 4.4. Implications and Future Directions

The results of this scoping review highlight the need for and importance of incorporating physical activity as a habit and lifestyle into the daily routine of university students. The role of universities could be to develop and incorporate physical activity into their academic offerings with combinations of endurance and aerobic training and the possibility of less intense offerings such as yoga or tai chi sessions. This offers students the opportunity to access physical activity and increase their motivation. This could complement other social interventions for mental well-being. The use of digital fitness apps and tools can be a crucial element in encouraging students to be active and especially to stay active by monitoring their stress levels.

### 4.5. Limitations of the Study

The limitations of the present study include the limited number of analyzed results and the exclusion criteria, which focused solely on stress-related studies without incorporating other mental health factors. Furthermore, the study relied on only two databases. Future research could expand its scope to explore broader aspects of mental well-being and concentrate on experimental interventions to better generalize findings and establish a comprehensive intervention protocol.

## 5. Conclusions

University students are a category of young adults who are highly exposed to stress and the possibility of developing mental ill health. In addition, university students are often highly sedentary due to the life transition they undergo, academic stress and pressure, the change in life away from their families, and the change in lifestyle. Physical activity is a powerful tool for improving the physical and mental well-being of university students. While moderate–vigorous physical activity tends to offer more immediate relief from stress, endurance training offers long-term benefits that contribute to general physical and mental well-being.

What is therefore recommended is an integrated approach that combines different forms of exercise as well as coping and emotional regulation strategies that could provide greater and more comprehensive benefits to students. The role of universities could use these findings to provide academic proposals aimed at student well-being in a holistic sense.

## Figures and Tables

**Table 1:** Summarizes the results grouped by study design.

Study Design	Authors
Experimental	Cai et al. [12], Flood et al. [13], Yan et al. [14], Zeng et al. [15], Martin et al. [16], Yang et al. [17], Lee et al. [18], Suwannakul et al. [19], Brown et al. [20], Jelleli et al. [21].
Quasi-experimental	Gao et al. [22], Johannes et al. [23], Chauhan et al. [24], Suguis et al. [25], Ming et al. [26], Herbert et al. [27], Donnelly et al. [28], Huckvale et al. [29].
Cross-sectional observational	Fruehwirth et al. [30], Byshevets et al. [31,32], Albikawi et al. [33], Stults et al. [34], Rongrong et al. [35], Shi et al. [36], Oftedal et al. [37], Zhu et al. [38], Teuber et al. [39], Kabiri et al. [40], Mu et al. [41], Howie et al. [42].
Longitudinal	Szmodis et al. [43], Lee et al. [18], Yan et al. [14], Teuber et al. [39], Reschke et al. [44]
Systematic Review	Donnelly et al. [28], Qi et al. [45], Huang et al. [46], Woodall et al. [47], Huckvale et al. [29], Barradas et al. [48].
Qualitative	Oftedal et al. [37], Stults et al. [34], Zou et al. [49], Khajavi et al. [50], Chauhan et al. [24], Barradas et al. [48].

## Appendix A

**Author**	**Title**	**Objectives of the Study**	**Design of the Study**	**Sample or Population/Study Included**	**Materials**	**Results and Outcomes**
Albikawi, Z. F. [33]	Perceived stress, physical activity, and insomnia of female nursing university students in Saudi Arabia: a cross-sectional study	Investigate female nursing students’ levels of perceived stress, practicing physical activity, and insomnia and examine related relationships	Cross-sectional study	290 students	Sociodemographic questionnaire, the perceived stress and international physical activity questionnaire, and the Bergen insomnia scale	Physical inactivity and use of the telephone before sleep increase the risk of insomnia; perceived stress is associated with reduced sleep quality.
Al-Wardat et al. [55]	Exploring the links between physical activity, emotional regulation, and mental well-being in Jordanian university students.	Connection between physical activity, emotional regulation, and mental health symptoms (depression, anxiety, and stress) in Jordanian university students	Cross-sectional study	416 students	Sociodemographic questionnaire, International Physical Activity Questionnaire (IPAQ-SF), Emotion Regulation Questionnaire (ERQ), Depression Anxiety Stress Scale (DASS)	High physical activity is associated with lower depression and anxiety; emotional expression affects improved mental well-being
Begdache et al. [56]	Dietary factors, time of the week, physical fitness and saliva cortisol: Their modulatory effect on mental distress and mood	Assess the effect of diet quality, in a population of different physical fitness, on saliva cortisol while accounting for mood during a peak day of the week and a weekend among men and women	Experimental study	48 students	Sociodemographic questionnaire, Mood and Anxiety Symptom (MASQ), Kessler Psychological Distress Scale (K10) and the General Nutritional Knowledge Survey (GNKS) questionnaires. Record dietary intakes for the three days of exercise session. Cycling sessions. Salivary cortisol level with sample before and after exercise session	High post-exercise cortisol associated with mood improvement; regular fitness reduces mental distress
Bhuiyan et al. [57]	Assessing the stress-buffering effects of social support for exercise on physical activity, sitting time, and blood lipid profiles	Test the hypothesized stress-buffering effects of social support on physical activity, sitting time, and blood lipid profiles	Cross-sectional study	537 students	Students enrolled in physical activity courses and exercises completed sociodemographic questionnaire, Perceived Stress Scale (PSS-4), modified version of the Social Support and Exercise Survey, Global Physical Activity Questionnaire (GPAQ) and measure of total cholesterol level, low-density lipoproteins (LDLs), and high density lipoproteins (HDLs)	Social support promotes moderate/vigorous physical activity and reduces sedentary behaviors; less stress associated with better fitness
Brown et al. [20]	PEAK mood, mind, and marks: A pilot study of an intervention to support university students’ mental and cognitive health through physical exercise	Impact of PEAK on exercise, mental and cognitive health, and implementation outcomes	Quasi-experimental pilot study (single-arm).	115 students	PEAK program involving exercise sessions, digital support, in-person activities, motivational videos, weekly differentiated exercises, neuroscientific focus on a single group	Increase in moderate–vigorous exercise, reduction in sedentary behaviors, improvements in mental well-being and cognitive abilities
Byshevets et al. [31]	The influence of physical activity on stress-associated conditions in higher education students	Substantiate the influence of physical activity on stress-associated conditions in higher education students. Higher education students at risk of emotional disorders under the influence of stress factors	Data-analysis based study with machine learning	1115 students	International Physical Activity Questionnaire-short version (IPAQ), Shcherbatykh Questionnaire (2002), Spielberger–Hanin Test (STAI), , Mississippi Scale for Assessment of Posttraumatic Reactions (civilian version)	Moderate physical activity reduces anxiety and stress; the models are predictive of detecting signs of PTSD and stress
Byshevets et al. [32]	Evaluation of emotional disorder risk in students with low physical activity levels under stressful conditions	Higher education students at risk of emotional disorders under the influence of stress factors	Cross-sectional study	573 students	Response of Ukrainian higher education students to the hostilities in the country	Students with low physical activity showed a higher risk of emotional distress during high stress conditions, especially among female students
Cai, L. [12]	Effect of physical exercise intervention based on improved neural network on college students’ mental health	Influence of physical exercise on mental health. The ways of employing physical exercise to improve the mental health of college students are presented in this study. Then, this paper proposes a physical exercise intervention based on an improved neural network (NN), which has an impact on the mental health level of college students, and the effectiveness of this model is verified by simulation experiments	Experimental study	Not specified	Physical intervention based on an improved neural network model (NN)	The intervention significantly improved mental health and reduced negative emotions caused by psychological stress
Chaabna et al. [58]	Physical activity and its barriers and facilitators among university students in Qatar: A cross-sectional study	Conduct a sex-specific examination of PA prevalence, barriers, and facilitators among university students in Qatar and a sex-specific assessment of other lifestyle and demographic factors associated with PA in this student population	Cross-sectional study	370 students	IPAQ-SF, Pittsburgh Sleep Quality Index, Perceived Stress Scale (PSS-4)	64.9% of students were physically active; major barriers included inadequate time and infrastructure, especially among women
Chauhan et al. [24]	Effect of yoga in medical students to reduce the level of depression, anxiety, and stress: Pilot study (Goodbye Stress with Yoga GSY)	Effect of yoga intervention on the level of stress, depression, and anxiety of medical student at the University of Pécs	Quasi-experimental pilot study	28 students	90-min yoga sessions once a week, following the GSY Goodbye Stress with Yoga Protocol for 10 weeks	Yoga significantly reduced depression (*p* = 0.019) and anxiety (*p* = 0.049), improving overall well-being
Donnelly et al. [28]	The effectiveness of physical activity interventions in improving higher education students’ mental health: A systematic review	Systematically review the evidence available regarding the impact of PA-related interventions to improve mental health and QoL outcomes in HE students	Systematic review	28 studies included	Systematic review on ProQuest, MEDLINE, Embase, CINAHL, SPORTDiscus e CENTRAL	Moderate–vigorous activity interventions such as Pilates and dance were most effective in improving mental health and quality of life
Fernández-Barradas et al. [48]	Physical activity and engagement coping: A key for stress-recovery in Mexican university students	Analyze the influence of physical activity and coping styles on recovery-stress state among Regular Physical Activity University Students (*n* = 67) and High-Performance University Athletes (*n* = 67) from a Mexican university	Comparative study	134 students	HPUA (High-Performance University Athletes) and RPAUS (Regular Physical Activity University Students) to compare the difference in coping strategies and recovery from stress using the Coping Strategies Inventory, the International Physical Activity Questionnaire (IPAQ), and the Recovery Stress Questionnaire	Athletes show greater resilience to stress; engaging coping and physical activity improve overall well-being
Flood et al. [13]	Development of a participation app–based intervention for improving postsecondary students’ 24-h movement guideline behaviors: Protocol for the application of intervention mapping	Develop and implement a theory-informed intervention intended to improve the movement behaviors and mental well-being of first-year postsecondary students	Intervention development and implementation protocol.	Not specified	App-based intervention (ParticipACTION) with content articles, push notifications, engagement badges, and resource packs	The app improved physical movement and mental well-being; the protocol serves as a model for other app-based interventions
Fruehwirth et al. [30]	Perceived stress, mental health symptoms, and deleterious behaviors during the transition to college	Associations between different sources of chronic perceived stress and deleterious behaviors (eating disorder symptoms, insufficient sleep, and insufficient vigorous physical activity) among first-year college students	Cross-sectional study	885 students	Surveys based on perceived stress and negative behaviors	Chronic stress increases negative behaviors such as eating disorders and sleep insufficiency
Gao et al. [22]	Effectiveness of aromatherapy yoga in stress reduction and sleep quality improvement among Chinese female college students: A quasi-experimental study.	Effectiveness of aromatherapy yoga intervention in reducing stress and improving sleep quality among Chinese female college students	Quasi-experimental study	89 students	Wilcoxon test, Mann–Whitney, questionnaires, yoga intervention with aromatherapy vs. traditional yoga with experimental group and control	Yoga with aromatherapy slightly improves sleep disturbances but not stress
Gubareva et al. [53]	Engaging in sports as a method to enhance the stress resilience of a student’s body	Evaluate the impact of sports activities on the adaptation of university students’ functional and psychophysiological systems to the stress induced by exams	Cross-sectional study	160 students	Physiological analysis with software, psychophysiological testing. Regular sports activities with sports and non-sports students	Sports students show greater resistance to stress during exams than non-sports students
Hachenberger et al. [52]	Investigating associations between physical activity, stress experience, and affective well-being during an examination period using experience sampling and accelerometry	To investigate the associations of physical activity, stress, and affective well-being in daily life by also considering objectively assessed physical activity	Mixed-method study	90 students	Accelerometry, experiential sampling, physical activity intervention during exam period. Without direct control group	Light physical activity reduces stress and improves emotional well-being
Herbert, C. [27]	Enhancing mental health, well-being and active lifestyles of university students by means of physical activity and exercise research programs	Physical activity and exercise interventions can help to promote the mental health of emerging adults such as university students	Literature review for research project	5 studies of interventions	Analysis of longitudinal data and interventions of aerobic physical activity without direct control group	Low/medium intensity physical activity improves mental health and stress perception
Hou Q., et al. [59]	Influences analysis of physical exercise on college students’ satisfaction and its psychological mechanism	Verify the impact of physical exercise on college students’ physical quality and personal satisfaction	Cross-sectional study	889 participants	Analysis of exercise-related personal satisfaction. Analysis on subjective well-being and psychological connotation	Exercise reduces stress and promotes personal satisfaction and mental well-being
Howie et al. [42]	Associations between physical activity, sleep, and self-reported health with burnout of medical students, faculty and staff in an academic health center	Assess the health status and health behaviors of medical students, faculty, and staff in an academic health center in the US, and examine the associations between behaviors, physical and mental health outcomes and burnout	Cross-sectional survey	2060 students	International Physical Activity Questionnaire (IPAQ), Pittsburgh Sleep Quality Index, DASS-21	Poor sleep and physical activity have been linked to high burnout. Good quality sleep and leisure-time physical activity significantly reduced anxiety, depression, and physical pain
Huang et al. [46]	Effectiveness of physical activity interventions on undergraduate students’ mental health: Systematic review and meta-analysis	Assessed the effectiveness of physical activity interventions on undergraduate students’ mental health	Systematic review and meta-analysis	59 studies included	Systematic review and meta-analysis	Moderate effectiveness in reducing depression, anxiety, and stress; study quality varied, indicating potential bias
Huang and Liang [51]	Effective forms of physical exercise to promote the health of college students	Raise effective alternatives for promoting physical exercise in college students	Literature review	Studies analyzed included 5056 students	Policy review	Public policies needed to improve physical and mental habits, reduce stress, and encourage physical activity
Huckvale et al. [29]	Protocol for a bandit-based response adaptive trial to evaluate the effectiveness of brief self-guided digital interventions for reducing psychological distress in university students: The Vibe Up study	Effectiveness of mindfulness, physical activity, sleep hygiene and an active control on reducing distress, using a multi-arm contextual bandit- based AI- adaptive trial method. Furthermore, the study will explore which interventions have the largest effect for students with different levels of baseline distress severity	Randomized trial protocol with AI	Not specified final number	Digital interventions (mindfulness, physical activity, sleep hygiene) vs. AI-driven adaptive methods control, DASS-21	All interventions reduced psychological distress; AI identified the most effective approach for specific symptoms
Jelleli et al. [21]	Examining the interplay between physical activity, problematic internet use and the negative emotional state of depression, anxiety and stress: Insights from a moderated mediation path model in university students	The aim of this study was to investigate the relationship between Problematic Internet Use (PIU), emotional states of stress, anxiety and depression, and the practice of physical activity among Tunisian students	Moderated mediation model analysis	976 students	Sociodemographic questionnaire, the Depression, Anxiety and Stress Scale—21 items (DASS-21), the International Physical Activity Questionnaire (IPAQ) and the compulsive internet use scale (CIUS)	Increased social interaction significantly reduced anxiety and improved stress management among active participants
Johannes et al. [23]	Relationship between psychosocial factors and physical activity among undergraduate students from a South African university	Determine the relationship between psychosocial factors and physical activity participation among undergraduate university students at a historically disadvantaged university (HDU) in South Africa	Cross-sectional studies	534 students	International Physical Activity Questionnaire—Short Form (IPAQ-SF), Depression, Anxiety and Stress Scale—21 (DASS-21), Physical Activity and Leisure Motivation Scale (PALMS), Perceived Social Support from Family and Friends Scale (PSS-Fa e PSS-Fr)	Social support and motivation are strongly correlated with higher levels of physical activity; stress and anxiety improve with regular exercise
Kabiri et al. [40]	Lower perceived stress among physically active elite private university students with higher levels of gratitude	Investigate the potential independent and interaction effects of physical activity and gratitude on perceived stress among undergraduates at elite private universities	Cross-sectional study	145 students	Study on gratitude and physical activity	Gratitude was associated with a significant reduction in perceived stress (*p* < 0.001); physical activity had no significant direct effects
Kan et al. [60]	Exploring the mediating roles of physical literacy and mindfulness on psychological distress and life satisfaction among college students	Explore the roles of physical literacy (PL) and mindfulness in mediating the impact of psychological distress on life satisfaction among college students in China	Cross-sectional study	653 students	Online survey and SEM analysis	Awareness and “physical literacy” positively mediate life satisfaction by reducing psychological distress
Khajavi et al. [50]	The effects of web-based education on health-promoting behaviors of first-year medical sciences students: A quasi-experimental study	The effects of web-based education on health-promoting behaviors of first-year medical sciences students: A quasi-experimental study	Quasi-experimental study (single-group pretest-posttest design).	185 students	Online educational program to promote healthy living. Almost experimental and educational videos sent via WhatsApp	Significant increase in responsibilities for health and stress management; minimal improvements in other areas
Lee et al. [18]	The effects of accumulated short bouts of mobile-based physical activity programs on depression, perceived stress, and negative affectivity among college students in South Korea: Quasi-experimental study	Investigate the effects of mobile-based PA programs delivered in the form of accumulated bouts on mental health indicators, including depression, perceived stress, and negative affectivity among healthy college students in South Korea	Quasi-experimental study	46 students	Short physical activity sessions (10 min, 2/day, 3 days/week)	Significant reduction in depression and stress in the experimental group; no improvement in the control group
Lepping et al. [61]	Physical activity, stress, and physically active stress management behaviors among university students with overweight/obesity	Associations between physical activity and stress management behaviors among students (18–35 years)	Randomized Controlled Trial (RCT)	405 students	Use of physical activity to manage stress among students with overweight/obesity. Accelerometers and stress questionnaires	Physical activity reduces perceived stress and improves active stress management with differences between races and levels of activity
Li et al. [62]	Epidemiological study of physical activity, negative moods, and their correlations among college students	Current status and correlation between physical activity and negative moods in college students	Cross-sectional epidemiological study	3711 students	IPAQ and DASS questionnaires	Low physical activity is related to depression and anxiety; increased intensity of exercise reduces negative symptoms
Li et al. [63]	Multidimensional impact of sport types on the psychological well-being of student athletes: A multivariate investigation	How competitive versus non-competitive sports affect Korean university students’ psychological well-being using a quantitative approach with SmartPLS 4 for multi-group analysis	Multivariate analysis	975 participants	Multivariate analysis with SmartPLS 4	Competitive sports improve mental endurance and stress management; non-competitive sports improve overall well-being
Li et al. [64]	The effect of exercise on academic fatigue and sleep quality among university students	Examine the prevalence and risk factors for academic fatigue among college students and its adverse effects on well being	Mixed-method study	864 students	Questionnaires (PSQI, Smith Well-being) and exercise interventions with intervention group	Exercise reduces academic fatigue and improves sleep quality
Lin et al. [65]	Effects of Qigong exercise on the physical and mental health of college students: A systematic review and meta-analysis	Systematic review and meta-analysis to evaluate the effects of Qigong exercise on the physical and mental health of college students	Systematic review and meta-analysis	16 studies included (RCTs)	Meta-analysis of 16 RCT	Qigong improves flexibility, cardiorespiratory endurance and reduces symptoms of anxiety and depression
Liu et al. [26]	Effects of physical activity on depression, anxiety, and stress in college students: The chain-based mediating role of psychological resilience and coping styles	Association between physical activity and negative emotions—specifically, depression, anxiety, and stress—in college students	Cross-sectional study with mediational analysis	1380 students	Stratified random sampling and multivariate statistical analysis (SPSS, PROCESS)	Physical activity reduces negative emotions through psychological resilience and positive coping
Liu et al. [66]	The way to relieve college students’ academic stress: The influence mechanism of sports interest and sports atmosphere	How university physical education fosters academic performance by influencing students’ sports interests, particularly in enhancing their psychological resilience to mitigate academic pressure	Cross-sectional study	574 students	SEM modeling and questionnaire analysis	Sports interest and environment improve psychological resilience, reducing academic stress
Martin et al. [16]	Yoga as a contemplative practice and its contribution to participatory self-knowledge and student retention: a scoping review of the first-year undergraduate student transition	Determine the extent of the current literature on the prevalence of yoga as a contemplative practice that contributes to student well-being and self-knowledge in the first-year transition from high school to university	Scoping review	17 studies included	Systematic review of 17 studies	Yoga improves emotional regulation and reduces stress during the passage of university life
Monserrat-Hernández et al. [67]	Academic stress in university students: The role of physical exercise and nutrition	Determine the relationship between the practice of physical exercise, eating patterns, and academic stress among university students	Cross-sectional study	742 students	Mediterranean Diet Score (MDS), Stress Manifestation Scale of the Students Stress Inventory (SSI), survey on physical activity frequency	Exercise is associated with lower levels of stress; Mediterranean diet without significant associations
Mu et al. [41]	Influence of physical exercise on negative emotions in college students: Chain mediating role of sleep quality and self-rated health	Relationship between physical exercise and negative emotions among college students, incorporating sleep quality and self-rated health (SRH) as mediators to analyze the pathway mechanism of how physical exercise affects students’ negative emotions	Cross-sectional study	30,475 students	Physical Activity Rating Scale-3 (PARS-3), the Depression, Anxiety, and Stress Scale—21 (DASS-21), the Pittsburgh Sleep Quality Index (PSQI), and the 12-Item Short Form Health Survey (SF-12)	77.6% of students engage in low-intensity physical activity
Oftedal et al. [37]	Changes in physical activity, diet, sleep, and mental well-being when starting university: A qualitative exploration of Australian student experiences	Relationship between physical exercise and negative emotions among college students, incorporating sleep quality and self-rated health (SRH) as mediators to analyze the pathway mechanism of how physical exercise affects students’ negative emotions	Qualitative study using focus groups	16 students	Online surveys and focus groups on the correlation between university transition and lifestyle change and levels of physical activity	Barriers to well-being include stress, cost of healthy food, and difficulty in prioritizing physical activity
Parakh et al. [68]	An empirical investigation of engineering students’ attitude towards sports and physical education, cultural activities and stress management	Explore young adult’s experiences of how starting university influenced their physical activity, diet, sleep, and mental well-being, and barriers and enablers to health behavior change	Cross-sectional study	270 students	Questionnaire of 50 questions to assess five factors that influence stress, current participation in sport, reasons. Participation and reasons for not participating in sports and reasons for participating in cultural events activities	Sports activities improve physical health, while cultural activities help to manage stress
Qi et al. [45]	Effects of Taichi on physical and psychological health of college students: A systematic review	(1) To measure the percentage of students who participate in sports and physical education. (2) To identify the reasons for engineering students to participate or not participate in sports and physical education. (3) To assess the stress level among the students.(4) To find out effect and sources of stress and give suggestions to overcome stress. (5) To understand the most important reasons for engineering students to participation in cultural events	Systematic review	22 studies included	Systematic review of 22 studies	Improvements in flexibility, balance, endurance and reduction in anxiety and depression
Qin et al. [69]	Effect of physical exercise on negative emotions in Chinese university students: The mediating effect of self-efficacy	Impact of physical activity on negative emotions among university students and the mediating influence of self-efficacy, aiming to furnish empirical insights and a theoretical framework to enhance and optimize the mental health of this population comprehensively	Cross-sectional study	5341 students	Physical Activity Rating Scale (PARS-3), the General Self-Efficacy Scale (GSES), and the Depression Anxiety Stress Scales (DASS)	Exercise reduces negative emotions with significant mediation of self-efficacy
Reschke et al. [44]	Examining recovery experiences as a mediator between physical activity and study-related stress and well-being during prolonged exam preparation at university	Role of recovery experiences as a mediator of the relationship between physical activity as one specific recovery activity and both study-related stress and well-being	Longitudinal study	56 students	Heidelberg Stress Index (HEI-STRESS) for stress measurement, Life Satisfaction and Study Scale (LSS), I-PANAS-SF for positive and negative affect, Recovery Experiences Questionnaire (REQ), physical activity logs (weekly hours)	The recovery experiences partially mediated the relationship between physical activity and stress reduction as well as improved well-being during exam preparation
Rongrong and Jian [35]	A study on the influence of a single bout of moderate-intensity exercise on processing bias towards emotional information of individuals with high psychosocial stress levels	Explore the impact of a single bout of moderate-intensity exercise on cognitive bias of individuals with high psychosocial stress levels	Experimental study	42 students	Moderate-intensity exercise, Word-Face Stroop Task, Memory Bias Task, Interpretation Bias Task, Perceived Stress Scale (CPSS)	Moderate-intensity exercise increased attention and memory bias toward neutral and positive emotional information, reducing psychosocial stress levels
Shi et al. [36]	Self-appreciation is not enough: Exercise identity mediates body appreciation and physical activity and the role of perceived stress	Explore the specific mechanisms of action between body appreciation and physical activity and provide a theoretical reference for promoting college students’ physical activity	Short-term longitudinal study	345 students	Longitudinal questionnaires on identity and perceived stress	Positive body perception predicts physical activity; exercise identity averages the positive effects of body perception, moderated by stress
Stults-Kolehmainen et al. [34]	Qualitative and quantitative evidence of motivation states for physical activity, exercise and being sedentary from university student focus groups	Examine postulates of the WANT model utilizing a mixed-methods approach	Mixed-methods study	17 students	Focus group, “CRAVE” scale for motivational states	Stress and boredom affect motivation for physical activity or sedentary activities; motivational states change rapidly and systematically
Suguis, J. E. [25]	Physical exercise and socio-emotional skills as predictors of academic productivity among college students	Relationship between physical exercise, socio-emotional skills, and academic productivity	Quantitative cross-sectional study	380 participants	Physical Activity Questionnaire (adapted), Socio-Emotional Skills Survey, Academic Productivity Questionnaire	Physical exercise (life enhancement, physical performance) and socio-emotional skills (self-awareness, decision making) significantly predicted academic productivity
Suwannakul et al. [19]	Effects of Surya Namaskar^®^ yoga on perceived stress, anthropometric parameters, and physical fitness in overweight and obese female university students: A randomized controlled trial	Compare the physical fitness, anthropometric measures, and perceived stress between the SN yoga training program group and the control group	Randomized Controlled Trial (RCT)	44 students (22 intervention group, 22 control group)	Randomized Controlled Trial (T-PSS-10, VO2max)	Yoga improves flexibility, muscle strength, VO2max, and reduces BMI and perceived stress
Szmodisal et al. [43]	Effects of regular sport activities on stress level in sporting and non-sporting university students	Analyze the relationship between regular sport activities, body parameters, cortisol level, perceived stress and the frequency of psychosomatic symptoms in male and female university students	Comparative cross-sectional study	200 students	Measurements cortisol salivary, perceived stress	Sports students have lower cortisol levels, less stress and better body compositions than non-sports
Teuber, M. [39]	Physical activity improves stress load, recovery, and academic performance-related parameters among university students: A longitudinal study on daily level	How PA affects university students’ stress load and recovery as well as their perceived academic performance	Longitudinal study	57 students	Effects of physical activity on stress, recovery and academic performance during home study with active breaks	Active breaks improve stress and academic performance; physical activity in leisure time improves recovery and reduces dysfunctional stress
Tshikovhele et al. [70]	The association of exercise and self-esteem among first-year students registered at a rural university in South Africa	Investigate the association between exercise participation and self-esteem among first-year students at a rural university in South Africa	Cross-sectional study	320 students	Regular physical exercise: at least 30 min, three times a week for two months or more. Non-regular: <3 times/week	Rosenberg Self-Esteem Scale (RSES)
Wang et al. [71]	Prospective association between 24-h movement behaviors and mental health among overweight/obese college students: A compositional data analysis approach	Associations of movement behaviors with mental health outcomes among overweight/obese college students using a compositional data analysis approach	Prospective cohort study	437 students	Motor behavior of 24 h and mental health in overweight/obese students	Moderate physical activity and sleep reduce depression, anxiety and stress; sedentary behaviors negatively related to mental health
Woodall et al. [47]	A scoping review of 20 years of college fitness/wellness courses	Literature review investigated CBFW course content, structure, and the effectiveness of courses or programs that sought to improve knowledge (K), perceived health/fitness (PHF), physical health (PH), and mental health (MH)	Scoping review	23 studies involving 8059 students	Systematic review of 23 articles (2003–2023)	Courses improve physical fitness, perceived stress and mental well-being; suggested expansion for mental health content
Yan et al. [14]	An 8-week peer health coaching intervention among college students: A pilot randomized study	Assess the effectiveness of a randomized, 8-week peer health coaching program on PA, nutrition, sleep, social isolation, and mental health, among college students	Pilot Randomized Controlled Trial (RCT)	52 students	RCT, weekly coaching vs. control group	Significant increases in vigorous activity and positive well-being; effective coaching for stress and physical activity goals
Yang, C. [17]	Activities research in the music art environment based on digital art analysis and multi-physiological signal anxiety recognition	Analyze the influence of activities in the music environment on college students’ self-control, psychological stress and the mediating effect of self-control, and provide alternative activity plans for college students’ mental health promotion	Experimental study	180 students	The SCL-90 test, general effectiveness scales, digital music analysis	Significant reduction in anxiety and depression; the musical environment improves psychological quality and is acceptable to students
Yue and Xiao [54]	Effects of moderate-intensity physical training on students’ mental health recovery	Explore the effect of moderate-intensity physical training on students’ mental health recovery	Experimental study	100 students	Moderate-intensity physical training with badminton: 30 min/session, focusing on both aerobic exercise and fun gameplay, Exercise-Induced Emotion Scale	Exercise of moderate intensity improved resilience to physical fatigue, increased emotional involvement, and mobilized positive emotions. Post-exercise calm remained stable
Zeng et al. [15]	The relationship between physical exercise and mobile phone addiction among Chinese college students: Testing mediation and moderation effects	Direct effects of physical exercise on MPA in college students and whether any detected association of physical exercise with MPA was mediated by self-control, rumination, and psychological distress and modulated by loneliness	Cross-sectional study	1843 students	General physical exercise rated by the Physical Activity Rating Scale-3 (PARS-3), evaluating intensity, frequency, and duration	Physical exercise reduces mobile phone addiction through improved self-control, decreased rumination, and lowered psychological distress. The mediation effects were moderated by loneliness with stronger effects for lonelier students
Zhang et al. [72]	The roles of exercise tolerance and resilience in the effect of physical activity on emotional states among college students	The aim of this study was to investigate the association between PA and negative emotional states and further determine the mediating effects of exercise tolerance and resilience in such a relationship			IPAQ-SFDepression, Anxiety, and Stress Scale—21 (DASS-21), Connor–Davidson Resilience Scale (CD-RISC), Exercise Intensity-Tolerance (PRETIE-Q), VO2max test	Physical activity reduces negative emotional states (depression, anxiety, stress) indirectly through improved exercise tolerance and resilience
Zhu et al. [38]	An analysis of the role of college students’ core self-evaluation in the relationship between extracurricular physical exercise and academic stress	Explore the role of college students’ core self-evaluation in the association between extracurricular physical exercise and academic stress, and to provide a reference and basis for effectively alleviating current college students’ academic stress	Cross-sectional study	1249 students	Extracurricular physical exercise: assessed by weekly duration and frequency during the semester, China College Student Mental Health Screening Scale, Core Self-Evaluation Scale, self-developed questionnaire	Extracurricular physical exercise reduces academic stress both directly and indirectly via core self-evaluation. Core self-evaluation partially mediates the relationship (21.9% mediation effect). Worries about exams and lagging behind peers were the most affected stress dimensions
Zou et al. [49]	Neural correlates of physical activity moderate the association between problematic mobile phone use and psychological symptoms	Examine the association between PMPU and psychological symptoms in late adolescents, along with the potential moderating effect of PA and neural basis by brain gray matter volume (GMV)	Cross-sectional study	251 students	Problematic Mobile Phone Use Scale (PMPU), International Physical Activity Questionnaire (IPAQ-C), Depression, Anxiety, and Stress Scale—21 (DASS-21), MRI-based voxel-based morphometry	Physical activity significantly moderated the relationship between problematic mobile phone use and psychological symptoms (depression, anxiety, stress). Neural correlates included gray matter volume changes in the insula and precuneus regions, which further mitigated depressive symptoms

## Appendix B

**Section**	**Item**	**PRISMA-ScR Checklist Item**	**Reported on Page #**
**Title**			
Title	1	Identify the report as a scoping review.	“Relationship between sedentary lifestyle, physical activity and stress in university students and their life habits: a Scoping Review with PRISMA Checklist (PRISMA-ScR)”
**Abstract**			
Structured summary	2	Provide a structured summary that includes (as applicable): background, objectives, eligibility criteria, sources of evidence, charting methods, results, and conclusions that relate to the review questions and objectives.	Review of the literature on the relationship between physical activity, sedentariness and stress among college students with regard to their lifestyles. The benefits of physical activity in reducing stress and improving mental health strongly present in the literature are highlighted, while identifying the lack of standardized intervention protocols.
**Introduction**			
Rationale	3	Describe the rationale for the review in the context of what is already known. Explain why the review questions/objectives lend themselves to a scoping review approach.	The prevalence of sedentary behaviors among college students poses risks to physical well-being, but special consideration needs to be given to the mental health of this population. This review synthesizes the literature that has emerged on how physical activity can be a means of reducing sedentariness and improving lifestyle by leading to significant reductions in stress and mental distress and promote well-being in this vulnerable population.
Objectives	4	Provide an explicit statement of the questions and objectives being addressed with reference to their key elements (e.g., population or participants, concepts, and context) or other relevant key elements used to conceptualize the review questions and/or objectives.	To explore the correlation between physical activity and stress among college students by analyzing their lifestyles and sedentary levels, identify benefits, and assess the presence of standardized intervention protocols in the literature.
**Methods**			
Protocol and registration	5	Indicate whether a review protocol exists; state if and where it can be accessed (e.g., a Web address); and if available, provide registration information, including the registration number.	This scoping review adheres to the Protocol for Scoping Review [11] using the Prisma Extension for Scoping Reviews Checklist
Eligibility criteria	6	Specify characteristics of the sources of evidence used as eligibility criteria (e.g., years considered, language, and publication status), and provide a rationale.	Studies included were open-access, scientific papers and journal papers in English, published between 2022 and 2024, focusing on university students aged 19–44.
Information sources	7	Describe all information sources in the search (e.g., databases with dates of coverage and contact with authors to identify additional sources), as well as the date the most recent search was executed.	The search was conducted on PubMed and Scopus between September and October 2024, identifying two search managers and one data-mining manager also responsible for resolving any ambiguities.
Search	8	Present the full electronic search strategy for at least 1 database, including any limits used, such that it could be repeated.	Search terms included: “physical activity OR physical exercise OR exercise AND university student OR college student AND stress OR cortisol”. Searches were limited to titles, abstracts, and keywords.
Selection of sources of evidence	9	State the process for selecting sources of evidence (i.e., screening and eligibility) included in the scoping review.	Two independent reviewers screened titles, abstracts, and full-text articles for inclusion based on predefined criteria.
Data charting process	10	Describe the methods of charting data from the included sources of evidence (e.g., calibrated forms or forms that have been tested by the team before their use, and whether data charting was done independently or in duplicate) and any processes for obtaining and confirming data from investigators.	Data were extracted using calibrated forms by two reviewers working independently, with disagreements resolved through consensus.
Data items	11	List and define all variables for which data were sought and any assumptions and simplifications made.	The variables researched were physical activity in correlation with stress or states of mental distress (excluding mental illnesses or disorders), sedentary levels, and lifestyle habits of the university population alone.
Critical appraisal of individual sources of evidence	12	If done, provide a rationale for conducting a critical appraisal of included sources of evidence; describe the methods used and how this information was used in any data synthesis (if appropriate).	A critical appraisal of the individual studies was not conducted, as this is a scoping review.
Synthesis of results	13	Describe the methods of handling and summarizing the data that were charted.	A summary of the results was made, creating macrocategories of the results obtained according to the study design analyzed, and from there the data needed to reach conclusions were extracted.
**Results**			
Selection of sources of evidence	14	Give numbers of sources of evidence screened, assessed for eligibility, and included in the review, with reasons for exclusions at each stage, ideally using a flow diagram.	The included results were 61 from 1208 results that were screened and selected following a first principle of exclusion of duplicates or ineligible papers according to the filters placed. Then 1091 papers that did not fit the inclusion criteria were excluded following the analysis of the abstracts, and finally 43 that were considered eligible in the first instance were excluded because they were not found to be relevant in the analysis of the full paper. This left 61 eligible results according to the following criteria: research area, target population, language, age group, and open access.
Characteristics of sources of evidence	15	For each source of evidence, present characteristics for which data were charted and provide the citations.	A table with exclusion criteria was provided in the paper specifying the reasons for exclusion. A figure with the division of macro areas according to the type of research design was provided in the results section. An appendix was created with the titles of each result, author, methods and instruments used, objectives and outcomes.
Critical appraisal within sources of evidence	16	If done, present data on critical appraisal of included sources of evidence (see item 12).	No compilation required
Results of individual sources of evidence	17	For each included source of evidence, present the relevant data that were charted that relate to the review questions and objectives.	All relevant information can be found in Appendix A, which contains the main outcomes of the studies considered.
Synthesis of results	18	Summarize and/or present the charting results as they relate to the review questions and objectives.	Physical activity consistently shows benefits for stress reduction and mental well-being in college students but with lack of standardized intervention protocols. Studies reports use of yoga, taichi, qigong, mindfulness, resistance training as major choices of intervention.
**Discussion**			
Summary of evidence	19	Summarize the main results (including an overview of concepts, themes, and types of evidence available), link to the review questions and objectives, and consider the relevance to key groups.	Physical activity consistently shows benefits for stress reduction and mental well-being in college students. Lifestyles in particular with the transition to college life particularly affect the change in physical activity levels and the increase in sedentariness. These factors, in addition to academic pressures, increase states of stress and mental unwellness. Regular physical activity programs, increased daily experience of movement in daily life, increased motivation to exercise or sports help to improve stress states, reduce sedentariness, and improve mental and physical well-being. However, the significant presence of studies highlights the lack of standardized intervention protocols limiting the generalizability of results and effective intervention. On the other hand, this leaves room for the cross-cutting nature of daily physical activity and exercise as always effective means of intervention for managing mental and physical well-being status and academic performance in such a fragile population.
Limitations	20	Discuss the limitations of the scoping review process.	The limitations of the present study include the limited number of analyzed results and the exclusion criteria, which focused solely on stress-related studies without incorporating other mental health factors. Furthermore, the study relied on only two databases. Future research could expand its scope to explore broader aspects of mental well-being and concentrate on experimental interventions to better generalize findings and establish a comprehensive intervention protocol.
Conclusions	21	Provide a general interpretation of the results with respect to the review questions and objectives, as well as potential implications and/or next steps.	College students face high levels of stress and mental health risks, compounded by a sedentary lifestyle due to life transitions, academic pressure, and lifestyle changes. Physical activity improves their physical and mental well-being: moderate-vigorous exercise provides immediate stress relief, while resistance training offers long-term benefits. An integrated approach combining different exercises and emotional regulation strategies is recommended. Universities should develop holistic programs to support student well-being.
**Funding**			
Funding	22	Describe sources of funding for the included sources of evidence, as well as sources of funding for the scoping review. Describe the role of the funders of the scoping review.	No external funding was received for the development of this paper.
	JBI = Joanna Briggs Institute; PRISMA-ScR = Preferred Reporting Items for Systematic reviews and Meta-Analyses extension for Scoping Reviews.		

## Appendix C

**Author**	**Eligibility Criteria Specified**	**Subjects Randomly Allocated**	**Allocation Concealed**	**Groups Similar at Baseline**	**Blinding of Subjects**	**Blinding of Therapists**	**Blinding of Assessors**	**Measures Obtained from >85% of Subjects**	**Intention-to-Treat Analysis**	**Between-Group Statistical Comparisons**	**Point Measures and Variability Data Reported**	**Total Score**
Stults-Kolehmainen et al. [34]	1	1	0	1	0	0	1	1	0	1	1	6
Begdache et al. [56]	1	1	0	1	0	0	0	1	1	1	1	6
Brown et al. [20]	1	1	0	1	0	0	1	1	1	1	1	7
Cai, L. [12]	1	1	1	1	1	0	1	1	1	1	1	9
Chauhan et al. [24]	1	1	1	1	1	1	1	1	1	1	1	10
Gao et al. [22]	1	1	1	1	0	0	0	1	1	1	1	7
Hachenberger et al. [52]	1	1	0	1	0	0	1	1	1	1	1	7
Huckvale et al. [29]	1	1	0	1	0	0	0	1	1	1	1	6
Khajavi et al. [50]	1	1	0	1	0	0	0	1	0	1	1	5
Lee et al. [18]	1	1	1	1	0	0	0	1	1	1	1	7
Lepping et al. [61]	1	1	0	1	0	0	1	1	0	1	1	6
Li and Chen [64]	1	1	1	1	0	0	1	1	1	1	1	8
Rongrong et al. [35]	1	1	0	1	0	0	1	1	0	1	1	6
Suwannakul et al. [19]	1	1	0	1	0	0	0	1	0	1	1	6
Yan et al. [14]	1	1	1	1	0	0	1	1	1	1	1	8
Yue and Xiao [54]	1	1	0	1	0	0	0	1	0	1	1	5
